# Immunoregulatory effects of Huaier (Trametes robiniophila Murr) and relevant clinical applications

**DOI:** 10.3389/fimmu.2023.1147098

**Published:** 2023-06-28

**Authors:** Hongrong Long, Zhongcai Wu

**Affiliations:** ^1^ Department of cardiac function, Hubei Cancer Hospital, Tongji Medical College, Huazhong University of Science and Technology, Wuhan, Hubei, China; ^2^ Division of Nephrology, Tongji Hospital, Tongji Medical College, Huazhong University of Science and Technology, Wuhan, Hubei, China

**Keywords:** Huaier, traditional Chinese medicine, immunoregulation, innate immunity, adaptive immune system, macrophages, dendritic cells, T lymphocytes

## Abstract

Huaier (Trametes robiniophila Murr) is a medicinal fungus of traditional Chinese medicine with more than 1000 years of history of clinical application. Its remarkable anticancer activities has led to its application in treating diverse malignancies. In recent years, the immunomodulatory effects of Huaier have been uncovered and proved to be beneficial in a plethora of immune-related diseases including cancer, nephropathy, asthma, etc. In this review, we comprehensively summarized the active components of Huaier, its regulatory activities on multifaceted aspects of the immune system, its application in various clinical settings as well as toxicologic evidence. Based on currently available literature, Huaier possesses broad-spectrum regulatory activities on various components of the innate and adaptive immune system, including macrophages, dendritic cells, natural killer cells, T and B lymphocytes, etc. Versatile immunologic reactions are under the regulation of Huaier from expression of damage-associated molecular patterns, immune cell activation and maturation to cell proliferation, differentiation, antibody production, expression of cytokines and chemokines and terminal intracellular signal transduction. Moreover, some modulatory activities of Huaier might be context-dependent, typically promoting the restoration toward normal physiological status. With excellent efficacy and minimal side effects, we foresee more extensive application of Huaier for treating immune-related disorders.

## 1 Introduction

The human immune system is constituted by a variety of lymphoid organs, cells and proteins that form a complex network of defense system against intruding pathogens and damaged cells. It can be classified into two interconnected types of response system: the innate immune system and the adaptive immune system (1). The innate immune system consists of cells like neutrophils, macrophages, dendritic cells (DCs), natural killer (NK) cells and related cytokines and enzymes that construct the first line of fast non-specific defense response (2). The adaptive immune system possesses high specificity and immunological memory that is critical in mounting potent, long-lasting and antigen-specific immune response. Adaptive immune system can be further divided into two distinct branches: humoral immunity and cell-mediated immunity. The former is primarily mediated by B lymphocytes that generate antigen-specific antibodies, while the latter involves T lymphocytes that execute cytotoxic killing, immunoregulation and facilitation of B cell activation (3). Well-operated immune system is crucial in maintaining body homeostasis, protecting against infection and elimination of malignant cells; however, derangement of the immune system underlies a constellation of human diseases, including autoimmune diseases (4), hypersensitivity (5), chronic inflammatory diseases (6), neoplastic diseases (7) and immunodeficiency (8). Thus, modulation of the immune system is essential for the treatment of various immune-related disorders, and it is an urgent task of clinical medicine to discover safe and effective drugs with broad-spectrum of immunoregulatory potentials.

Currently available immunomodulatory drugs exhibit limited therapeutic windows, drug dependance and significant side effects. Consequently, research efforts have increasing focused on the exploration of natural compounds for drug development, with traditional Chinese medicine (TCM) serving as valuable treasury. Trametes robiniophila Murr (Huaier), an officinal fungus grown on trunks of trees such as Sophora japonica, has a record of clinical application in TCM for more than 1000 years (9). In recent years, potent antitumor effects of Huaier have been noted in various neoplastic diseases including breast cancer, hepatocellular carcinoma, lung cancer and gastrointestinal cancer (10), etc. Besides, strong and broad-spectrum immunoregulatory effects have also been discovered that not only contribute to its antitumor activity but also show efficacy in the treatment of a cluster of immune-related disorders comprising allergic (11), autoimmune (12) and infectious diseases (13). Nonetheless, comprehensive review of the immunomodulatory effects of Huaier and its related clinical implications have not been conducted thus far. To address this gap, we conducted a comprehensive review by searching for eligible literature on PubMed using the keywords “Huaier”, “Trametes robiniophila Murr”, or “Huaiqihuang”, from inception till October 15th, 2022. Literature in reference lists were also screened for eligibility. We collected literature of laboratory research, clinical research and reviews involving Huaier or Huaier-derived preparations in English or Chinese. All search results were manually inspected based on their titles and abstracts, and full texts were retrieved if they were considered relevant. Then, reported therapeutic effects of Huaier were extracted from each article, classified and summarized in tabulated form. Any discrepancies between reviewers were resolved through discussion to reach consensus. A total of 164 relevant articles were retrieved for our study. In this review, we presented the constituents and active components of Huaier, summarized the up-to-date evidence of the immune regulatory functions of Huaier-derived reagents, and further discussed their differential efficacy and implications in a variety of clinical settings.

## 2 Components of Huaier

### 2.1 Crude extracts of Huaier

#### 2.1.1 Aqueous extract of Huaier

As most medicinal fungi, the ingredients of Huaier are complex and include polysaccharides, proteins, ketones and alkaloids, etc. Researches for identifying effective components are still on the way. Among them, polysaccharides and proteoglycans are considered to be the major bioactive substances (14). Aqueous extraction is the most adopted method to produce Huaier preparation (15), which yields crude aqueous extract of Huaier containing 41.5% of polysaccharides, 12.93% of amino acids and 8.72% of water (16). Detailed constituents of Huaier extract include 6 monosaccharides and 18 amino acids that are listed in **Table 1** (26, 27).

**Table 1:** Constitutions of Huaier and Huaier-derived preparations.

Substance	Constitutions	MW (kDa)	Reference
Huaier aqueous extract	41.5% carbohydrates+12.93% amino acids+8.72% water Amino acids: Asp, Thr, Ser, Glu, Pro, Gly, Ala, Cys, Val, Met, Ile, Leu, Tyr, Phe, Lys, His, Trp, Arg Monosaccharides: fucose, arabinose, xylose, mannose, galactose, glucose	/	(9, 10)
Huaier n-butanol extract (HBE)	51.4% flavonoids	/	(13)
TPG-1	43.9% carbohydrates + 41.2% amino acids Amino acids: Asp, Thr, Ser, Glu, Gly, Ala, Cys, Val, Met, Ile, Leu, Tyr, Phe, Lys, His, Arg, Pro Monosaccharides: glucose, xylose, arabinose, rhamnose, galactose, mannose	~55.9	(17)
HP-1	/	~30	(18, 19)
TP-1	93.2% carbohydrate, free of proteins and uronic acids Monosaccharides: mannose, galactose, rhamnose, glucose, arabinose (3.1:2.2:2.0:1.1:0.5 in molar ratio)	~2300	(20, 21)
W-NTRP	96.5% carbohydrate, free of proteins and nucleic acids Monosaccharides: galactose, arabinose, glucose (4.2:2.5:0.7 in molar ratio)	~25	(2)
SP1	94.35% carbohydrate, free of proteins and uronic acids Monosaccharides: galactose, arabinose, glucose (3.1:1.1:1.1 in molar ratio)	~56	(22, 23)
HQH	Trametes robiniophila Murr (Huaier), Lycium barbarum and Polygonatum sibiricum	/	(24, 25)

#### 2.1.2 Butanol extract of Huaier

In order to retain more bioactive components with poor solubility or heat sensitivity, an alternative method uses alcoholic solvents to obtain the Huaier n-butanol extract (HBE, NEH or TRMBE), which includes flavonoids as the major component with a proportion of 51.4% in the final product. *In vitro* and *in vivo* experiments demonstrated beneficial health effects of HBE in combating tumor growth, metastasis and modulating immune function while *in vivo* toxicity remains negligible even in high dose (17, 20–22, 28).

### 2.2 Purified homogenous Huaier-derived compounds

Further efforts have been dedicated to purify homogenous bioactive polysaccharides or proteoglycans from crude extracts of Huaier (9, 18, 19, 23, 24, 29). **Table 1** displays summary information of known purified ingredients from Huaier, which includes TPG-1, TP-1, HP-1, W-NTRP and SP1. The immunoregulatory activities of each substance is summarized in **Table 2**.

**Table 2:** Immunoregulatory effects of Huaier and its derivatives.

Substance	Immunoregulatory activities
Huaier aqueous extract	• Hemopoiesis: Reduce incidence of myelosuppression • DAMP: ↑Production by tumor cells • Lymphocytes: ↑Increase CD3+ and CD4+ T cells; ↑CD4+/CD8+ ratio ↑Th1 differentiation in cancer ↓Th1,↓Treg, ↑Th17 in ITP • NK cells: ↑Increase NK cells; ↑NK activity • DC cells: ↑Promote maturation ↑DC-induced T cell proliferation & Th1 differentiation • Macrophages: ↓Proliferation and infiltration in tumor ↑Promote M1 polarization; ↑Promote phagocytosis ↓Suppress motility; ↓MΦ-induced angiogenesis
Huaier n-butanol extract (HBE)	• Lymphocyte: ↓PD-1+ CD8+T cell • NK cells: ↑Increase • Macrophage: ↓MΦ in spleen • MDSCs: ↓PMN-MDSC • Cytokines in blood: ↓IL-6, IL-10, TGF-β; ↑IFN-γ
TPG-1	• Leukocytes: ↑Infiltration in tumor • Macrophages: ↑M1 polarization; ↑Production of NO, TNFα, IL-6 ↑TLR4/NF-κB and MAPK
TP-1	• Immune organs (Thymus and Spleen): ↑Weight • Lymphocytes: ↑Proliferation; ↑CD3+ T cells and CD4+ T cells ↓CD8+ cells • NK cells: ↑Quantity in spleen
W-NTRP	• Lymphocyte: ↑Proliferation • Macrophage: ↑M1 polarization; ↑Phagocytosis
HQH	• Lymphocytes: ↓Infiltration in asthma ↑CD3+ T cells and CD4+ T cells; ↑Increase CD4+/CD8+ ratio ↓CD8+ T ↑Th1/Th2 ratio in asthma; ↓Th17/Treg balance in asthma • Antibody: ↑IgA, IgM, IgG; ↓IgE • Macrophages: ↓Infiltration; ↑Phagocytosis • Neutrophils: ↓Infiltration in asthma • Eosinophils: ↓Infiltration in asthma

#### 2.2.1 TPG-1

TPG-1 is a Huaier-derived proteoglycan with a molecular mass of around 55.9kDa composed of 43.9% of carbohydrates and 41.2% of amino acids. It has been shown to possess strong immuno-potentiating and antitumor effects as it stimulated the secretion of tumoricidal cytokines TNF-α and IL-6 by activating the TLR4- and MAPK-mediated NF-κB signaling pathways in macrophages and markedly restricted the growth of xenograft hepatoma in nude mice (23).

#### 2.2.2 TP-1

A purified colorless polysaccharide of Huaier called TP-1 was proved to be a homogenous compound of around 2300kDa containing 93.2% carbohydrates and free of proteins and uronic acid. Gas chromatography analysis demonstrated TP-1 to be composed of mannose, galactose, rhamnose, glucose, and arabinose at molar ratios of 3.1:2.2:2.0:1.1:0.5. Biological experiments confirmed the antitumor, anti-angiogenic and immune stimulating effects of TP-1 (29–31).

#### 2.2.3 HP-1

Similar chromatographic method yielded another purified homogenous polysaccharide named HP-1 that was determined to be water soluble with a molecular weight of around 30kDa. Anticancer, antioxidative and anti-EMT effects of HP-1 have been observed by scholars, which implies its potential therapeutic values in the treatment of cancer and prevention of chemotherapy-induced nephrotoxicity (32, 33).

#### 2.2.4 W-NTRP

Sun et al. isolated a homogenous neutral water-soluble polysaccharide from Huaier and named it as W-NTRP. The product was determined to be about 25kDa that contains 96.5% of total carbohydrates and free of proteins and nucleic acids. Galactose, arabinose and glucose were identified as basic constituents of W-NTRP with a relative molar ratio of 4.2:2.5:0.7. The selective tumoricidal and immune-stimulating activities render it a potential therapeutic agent against cancer (9).

#### 2.2.5 SP1

SP1, a Huaier-derived homogenous polysaccharide, was determined to contain 94.35% total carbohydrate content with uronic acid and protein contents undetectable. Gas chromatography analysis revealed that galactose, arabinose and glucose in relative molar ratios of 3.1:1.1:1.1 constitute this 56kDa polysaccharide. *In vitro* and *in vivo* experiments demonstrated sound anti-cancer activities in models of hepatocellular carcinoma and breast cancer (24, 34).

## 3 Immunomodulatory activities of Huaier

Numerous modulatory activities of Huaier involving various aspects of the immune system have been reported in published literature due to its extensive application and complicated ingredients. In conducting literature review, we noted that Huaier in some respects produces context-dependent rather than invariable regulatory effects according to the underlying pathophysiology. Briefly speaking, Huaier usually produces regulatory activities that promote the regression of abnormal immune status toward normal physiology. Furthermore, Huaier possesses negligible toxic effects on healthy cells and organs (35). These incredible features bring about broad scale of therapeutic benefits which makes Huaier an effective treatment in a multitude of clinical settings. In the following discussion, we systemically summarized the detailed regulatory effects of Huaier on multifaceted aspects of the immune system, and opposite activities of Huaier might be mentioned due to differences of clinical situations. Brief summary of the specific applications of Huaier in different diseases will be made at the last part of this manuscript.

### 3.1 Effects of Huaier on hemopoiesis

As an essential step that generates various progenitor cells, hemopoiesis is a prerequisite for the construction of intact immune system. However, in patients receiving chemotherapeutic anticancer agents, myelosuppression is not a rare complication leading to immunodeficiency, leukopenia and hypochromia. However, adjuvant Huaier treatment has been observed to rehabilitate hemopoietic function and significantly reduce the incidence of myelosuppression in patients with breast cancer, indicating the potential clinical value of Huaier in reconstructing immune function in immunodeficient patients (36, 37).

### 3.2 Effects of Huaier on innate immunity

The innate immune system forms the first line of defense against harmful pathogens by performing immune screening, antigen presentation and induction of adaptive immune responses. A variety of innate immune cells and cytokines participate in this process, and dysregulation of any elements of the innate immune system could result in immunodeficiency, severe infection, persistent chronic inflammation, allergic diseases or failed immunosurveillance against tumor cells (38, 39). Huaier showed numerous modulatory activities on many important participants of the innate immune system, including macrophages, natural killer (NK) cells, dendritic cells (DCs) and so on.

#### 3.2.1 The effects of Huaier on DAMP expression and release

The innate immune system accurately responds to invading pathogens or tissue lesions by recognizing evolutionarily conserved pathogen-associated molecular patterns (PAMPs) or damage-associated molecular patterns (DAMPs) with cognate receptors, which leads to the ensuing leukocyte infiltration and immunological activation. As a result, the expression of DAMPs acts the initiator of immunological reaction that participates in the pathogenesis of a plethora of inflammatory and neoplastic diseases (40, 41). It has been found that Huaier and Huaier-derived preparation interfere with the expression of DAMPs in different clinical settings.

Immunogenic cell death (ICD) has been proposed to play a pivotal role in cancer prevention and elimination. It has been shown that DAMPs together with their interacting receptors are involved in the process of ICD and activation of anticancer immunity. Huaier has been found to boost the expression and release of DAMPs, such as surface calreticulin (CRT), secreted ATP and passively released HMGB1, in several cell lines of triple negative breast cancer (TNBC). The enhanced DAMP exposure facilitated the maturation of DC cells and augmented DC-induced T cell response, which not only resulted in improved efficacy of Huaier-treated cancer cell vaccine but also mediated delayed tumor growth *via* oral administration of Huaier (42).

Acute kidney injury (AKI) is a heterogenous clinical disorder that primarily results from ischemia, hypoxia, nephrotoxicity or ureteral obstruction. Inflammation represents a pivotal component of AKI that exacerbates renal injury, and the expression of certain DAMPs such as HMGB1 contributes to the initiation and persistence of inflammation. HQH, the compound preparation of Huaier, was discovered to protect against cisplatin-induced nephrotoxicity by reducing the release and nuclear-cytoplasmic translocation of HMGB1, inactivate the downstream TLR4/NF-κB signaling pathway and ultimately result in markedly alleviated renal injury, which was comparable to the effects of dexamethasone (43).

#### 3.2.2 Effects of Huaier on macrophages

Macrophages are a group of functionally diverse and widely-distributed cells that paly an integral part in innate immunity. They selectively exert a multitude of functions during homeostasis and inflammation, including phagocytosis, antigen presentation, cytokine production and so on. As a kind of cells with high plasticity, macrophages are deemed to convert into two distinct subsets with opposite properties according to environmental cues: a) classically activated/M1-polarized iNOS-expressing macrophages that mainly play pro-inflammatory roles by secreting cytokines such as TNF-α, IL-1β, IL-6 and IL-12; b) alternatively activated/M2-polarized Arg1-expressing macrophages that primarily exert anti-inflammatory effects by producing cytokines like IL-10 and TGF-β. Dysregulated macrophage proliferation or polarization are involved in numerous pathological situations (44–46).

Huaier has been discovered to affect multifaceted aspects of macrophages in various diseases, including macrophage infiltration, motility, polarization, cytokine production, phagocytosis and pro-angiogenic activities. Reduced macrophage infiltration following the administration of Huaier has been reported in most situations, such as tumor tissues (47), spleens of cancer-bearing mice (17), kidneys from adriamycin-induced nephrotic rats (48) and bronchoalveolar lavage fluid (BALF) of asthmatic mice (49), indicating suppressed inflammation and macrophage-mediated tumorigenesis. The reduced macrophage infiltration is probably owing to suppressed macrophage motility by Huaier treatment, as Huaier not only possesses a direct inhibitory effect on macrophage motility but also reduces the expression of chemotactic factors like CSF-1, GM-CSF and VEGF (47). However, contrary observation has also been reported in one study that Huaier increased the number of CD68+ macrophages in N-nitrosodiethylamine-induced rat hepatic carcinoma (50). Unexpectedly, decreased fibrotic nodules, reduced atypical cells, upregulated IL-2R+ cells and NK cells have also been claimed in parallel to the increasement of macrophages in Huaier-treated model group, and the author speculated greater retention of Kupffer cells in better-preserved hepatic sinusoids in mice with mild histopathological changes. This observation contradicts with the prevailing theory that macrophages usually participate in carcinogenesis, and robust studies are in need to verify this experiment.

Skewed polarization of macrophages is implicated in neoplastic diseases as M1 macrophages often display antitumor effects while M2 macrophages usually facilitate tumor initiation and progression. Based on the currently available publications that we collected (9, 23, 47), consistent results suggested that Huaier suppresses M2 polarization and promotes M1 polarization in various neoplastic diseases that probably mediated by the activation of the TLR4-NF-κB/MAPK signaling pathway. Furthermore, the enhanced secretion of pro-inflammatory cytokines from Huaier-induced M1 macrophages might account for a great deal of Huaier-produced antitumor effects, as *in vitro* experiments only demonstrated weak direct tumor suppressive activity of the Huaier-derived proteoglycan TPG-1, whereas the IL-6 and TNF-α rich supernatant of TPG-1 treated macrophages showed a much stronger inhibiting effects on tumor growth (23).

Enhanced phagocytic activity is regarded as a hallmark of macrophage activation. Pre-treatment of Huaier extract produced dose-dependent reinforcement of the phagocytosis of RAW264.7 macrophage cell line (47). The stimulatory effect also remains valid by *in vivo* HQH treatment as enhanced phagocytic activities were observed in BALF-derived macrophages of HQH-treated asthmatic mice, which showed positive correlations with blood and BALF IFN-γ levels (51). The Huaier-derived polysaccharide W-NTRP might be responsible for this phagocytic stimulatory effect as it effectively potentiated the pinocytic activity of mouse primary peritoneal macrophages *in vitro*, especially at the concentration of 100 μg/ml that produced the maximum effect (9).

Macrophages are able to promote angiogenesis by secreting cardinal pro-angiogenic factors VEGF, MMP2, and MMP9 that are essential in regulating endothelial mitogenesis, apoptosis, vascular permeability, cell migration and vascular matrix remodeling. Under the context of neoplastic diseases, the pro-angiogenic activities of infiltrating macrophages bring about detrimental effects that facilitate tumor growth, invasion and metastasis. Huaier was discovered to suppress the pro-angiogenic effects of macrophages by reducing the production of aforementioned pro-angiogenic factors, which significantly attenuated micro-vessel density (MVD) in mouse xenograft tumor tissues (47).

#### 3.2.3 Effects of Huaier on dendritic cells

Dendritic cells (DCs) are professional antigen presenting cells that play key roles in activating naïve T cells and eliciting cell-mediated immune responses (52). The maturation of DC is a vital step in potentiating its antigen presentation ability (53). Heterogenous subsets of DCs exert distinct immunoregulatory activities that mediate either immuno-activation or immuno-tolerance and promote different fates of CD4+ T helper cells (54). Several signaling pathways are implicated in the survival and maturation of dendritic cells, such as the MAPK signaling pathway (p38, JNK and ERK) and the PI3K/Akt pathway (55, 56).

Huaier extract was discovered to elicit extensive changes in several lines of the aforementioned signaling pathways, such as down-regulation of the phosphorylation of p38, elevation of both total JNK and p-JNK, and up-regulated expressions of PI3K, Akt and p-Akt. Furthermore, as mentioned in previous section, Huaier could also indirectly activate DCs by promoting enhanced DAMP expression by tumor cells, which renders efficacy of Huaier-treated tumor vaccine in inhibiting tumor growth. Consequently, Huaier potently boosted the expression of co-stimulatory molecules CD40, CD86 and MHC II, increased secretion of the pro-inflammatory cytokines IL-1β and IL-12 and repressed phagocytic activity of DCs, which led to subsequent DC-facilitated T cell proliferation and Th1 differentiation. As a result, DCs and antitumor CD4+ and CD8+ T cells were observed to accumulate in Huaier-treated 4T1 breast cancer-bearing mice, together with restrained tumor growth and improved general conditions (42, 53).

#### 3.2.4 Effects of Huaier on natural killer cells

Natural killer (NK) cells are important effector killer cells of the innate immune system that play indispensable roles in antiviral and anticancer reactions (57, 58). Infiltrated NK cells in tumor microenvironment (TME) are positive indicator for good prognosis, while compromised NK cell activities are related to immunodeficiency. NK cell-targeted therapies are promising in the treatment of various diseases (59, 60).

Treatment with Huaier or Huaier-derived compounds not only increased the quantity but also raised the activity of NK cells. In hepatoma cell H22-inoculated mouse model, the administration of the Huaier-derived polysaccharide TP-1 significantly expanded the population of splenic NK cells (29). In patients receiving postoperative or neoadjuvant chemotherapy, it has also been observed that co-administration of Huaier granule markedly elevated the proportion or activity of peripheral blood NK cells (37, 61–64). The inhibitory activity of NK cells on hepatocellular carcinoma (HCC) cell line MHCC97-H was augmented by the treatment of Huaier polysaccharide at the concentration of 100 μg/ml, which showed further synergistic effects with depletion of the AEG-1 gene in tumor cells (65). Moreover, it has been shown that the synergistic anticancer effects of Huaier n-butanol extract (HBE) and the chemotherapeutic agent 5-fluorouracil (5-FU) is mainly mediated by expanding tumoricidal effectors like NK cells in TME, and the depletion of NK cells completely abolished the beneficial effects of the combination therapy (17). The effective reinforcement of anticancer immunity by the combination therapy is probably achieved by depleting tumor-infiltrating myeloid-derived suppressor cells (MDSCs) or modulating expression profile of tumor-related immune cytokines, wherein the immunosuppressive cytokines IL-6, IL-10 and TGF-β were reduced and the immune-stimulating cytokine IFN-γ was elevated by the application of the combined therapy.

#### 3.2.5 Effects of Huaier on neutrophils

Neutrophils are generally considered as the first responder to pathological insults that participate in the acute phase of inflammation (66). Substantial dysfunction of neutrophils leads to severe immunodeficiency and even mortality, whereas excessive activation and persistent infiltration of neutrophils cause tissue damage and sustained inflammation (67). HQH, the Huaier-derived compound preparation, showed efficacy in ameliorating the inflammatory status of the airway tissue in OVA-induced asthma model mice, wherein both tissue-infiltrated and migrated inflammatory cells in BALF were markedly reduced including neutrophils (49). However, this observation is more likely to be a secondary outcome of corrected T helper cell balance rather than a sequela of direct modulation by HQH, as the reduced Th17 subset and impeded IL-17 production would impose less chemoattractant effect on neutrophil recruitment.

#### 3.2.6 Effects of Huaier on eosinophils

Eosinophils are granulated immune cells of the myeloid origin that are closely involved in helminth infection and anaphylactic diseases (68, 69). As a TCM, Huaier has also been proved to regulate eosinophil activity. The asthma ameliorative property of HQH has been suggested to relate to its ability in reducing eosinophil infiltration in airway mucosa and in BALF lavage (49).

### 3.3 Effects of Huaier on adaptive immunity

The adaptive immunity renders specificity and plasticity to the immune system that are characterized by antigen-specific recognition, immune memory and strengthened response to repeated insult. T and B lymphocytes are the fundamental executors of adaptive immunity carrying particular antigen-specific receptors for accurate recognition (38). Appropriate modulation of the adaptive immune system is essential in treating a plethora of immune-related disorders, and Huaier displays good bioactive properties in this regard.

#### 3.3.1 Effects of Huaier on T cells

T cells serve as the central part of the adaptive immune system that carry out cell-mediated immunity, orchestrate innate immune reactions and activate B cell response. To fulfil these complicated tasks, T cells differentiate into functionally diverse types and subtypes that form a hierarchical structure. Briefly, effector T cells can be classified into CD4-bearing T helper (Th) cells or CD8-bearing cytotoxic cells (1, 38, 70). Heterogenous activated CD4+ Th cells can be subdivided into functionally diverse groups including Th1, Th2, Th17 and Treg cells that cross-regulate the activity of others to reach dynamic balanced statuses (71). Huaier showed broad-spectrum modulatory activities on various aspects of T cells from proliferation and cytokine production to differentiation and fate determination, which makes Huaier a promising agent with potent immune-modulatory activities for the treatment of a plethora of diseases.

Generally, Huaier has been reported to stimulate T cell proliferation and increase the quantity of T cells in peripheral blood and local tissues. Clinical studies revealed that Huaier increased the proportion of CD3+ and CD4+ T cells in peripheral blood in cancer patients receiving chemotherapy (37, 61–63, 72, 73); HQH, the compound preparation of Huaier, has also been claimed to raise serum levels of CD3+ and CD4+ T lymphocytes in pediatric patients suffering primary nephrotic syndrome (74) and severe Mycoplasma pneumonia (13). Elevated peripheral blood CD4+ and CD8+ T cells by the administration of Huaier polysaccharides were recapitulated in rat model simulating HCC recurrence after liver transplantation (75). Splenocyte proliferation assay provided direct evidence by revealing that both W-NTRP and TP-1, two of the Huaier-derived polysaccharides, boosted the proliferation of ex vivo lymphocytes from experimental mice either alone or in combination with the T-cell mitogen ConA or B-cell mitogen LPS (9, 29). IL-2, also known as T cell growth factor, is an essential product of activated T cells that works in an autocrine fashion by binding with T-cell expressed IL-2R. It has been noted in cancer that Huaier increased both IL-2 level and IL-2R-expressing cells indicating reinforced cell-mediated immunity (29, 50, 73). Besides, the stimulation of Huaier on T cell proliferation could be indirectly mediated by promoting the maturation and activation of dendritic cells to potentiate their T cell stimulatory abilities (42, 53). Furthermore, TP-1 increased the relative weights of spleen and thymus, two important lymph organs in charge of T cell development and lymphocyte storage, respectively. Splenic CD4+ T cells also increased significantly following Huaier administration in tumor-bearing mice leading to marked elevation of CD4+/CD8+ ratio (29). In chemically-induced malignant transformed tissues as well as in xenograft tumor tissues, Huaier treatment significantly increased CD4+ T cells (53), CD3+ T cells and CD8+ T cells (42) that played indispensable roles in mounting anticancer responses. Nonetheless, in rare situations the enhancement of T cell infiltration by Huaier treatment might cause unwanted consequence, as reported in mouse heterotopic heart transplantation model that increased CD8+ T lymphocyte infiltration induced by Huaier aggravated tissue damage and acute rejection (76). In addition, evidence suggested differential effects of Huaier on normal and malignant lymphocytes, and application of Huaier or HQH suppressed the growth of acute T cell leukemia cell line Jurkat cells and B cell precursor leukemia cell line Nalm-6 cells (77, 78).

It should be noted that Huaier exhibits differential regulations on CD4+ and CD8+ T cells, thereby adjusts the balance of CD4+/CD8+ ratio. To the best of our knowledge, currently available studies reporting the effects of Huaier or HQH on CD4+/CD8+ balance demonstrated consistent trends of upregulation (13, 29, 37, 61–63, 73–75, 79). Evidence thus far unanimously suggested stimulatory effects of Huaier or HQH on CD4+ T lymphocytes as mentioned previously, except for one study claiming that Huaier suppressed anti-CD3 and anti-CD28-induced PBMC activation and reduced IL-2 production of immune thrombocytopenia (ITP) patients (77). This unusual exception might be owing to the pathogenic predisposition of T cells to be easily activated in ITP patients. However, conflicting results are frequently seen in terms of the regulatory effects of Huaier on CD8+ T cells. The Huaier-derived polysaccharide TP-1 reduced the quantity of splenic CD8+ T cells in tumor-bearing mice (29). In mouse model of Echinococcus granulosus infection, oral intake of Huaier extract markedly decreased CD8+ T cells in peripheral blood of infected animals (79). For clinical studies, co-administration of Huaier or HQH as adjuvant therapy has been reported to reduce CD8+ T lymphocytes in advanced breast cancer patients (73), pediatric patients with primary nephrotic syndrome (74) and severe Mycoplasma pneumonia (13). On contrary, several animal experiments claimed that oral administration of Huaier increased CD8+ T lymphocytes in peripheral blood of a rat model of primary hepatocellular carcinoma (75) and in heart allografts of a mouse heterotopic transplantation model (76). Besides, one study indicated that the proportion of tumor-infiltrating PD1+ cells among CD8+ T lymphocytes decreased significantly by combined treatment of the Huaier butanol extract TRMBE and the chemotherapeutic agent 5-FU in mice bearing xenograft tumors, suggesting favorable enhancement of CD8+ T lymphocytes to resist apoptotic signals (17). In a word, Huaier generally tilts the balance of CD4+ and CD8+ cells toward the side of CD4+ Th cells either by producing greater stimulation on CD4+ T cells, or by simultaneously activating CD4+ cells and suppressing CD8+ cells.

Apart from the balance of CD4+ and CD8+ cells, the predominant Th cell subset also profoundly influences the overall immune status and participates in the initiation and resolution of related diseases. Huaier has been discovered to promote the balance of Th subsets by regulating both the Th1/Th2 and the Treg/Th17 cell ratios. However, inconsistent regulatory activities have been noted depending on the clinical contexts. The most comprehensive evaluation of the effects of Huaier on Th cell differentiation has been conducted in ovalbumin-induced asthma models, wherein HQH corrected the balances of both Th1/Th2 and Treg/Th17 cells, transcriptionally elevated the expression of Th1-driven transcription factor T-bet and Treg-driven factor Foxp3 while suppressed the Th2-driven Gata-3 and Th17-driven RORγt, functionally enhanced the levels of Th1 cytokine IFN-γ and Treg cytokine IL-10 while inhibited Th2 cytokines IL4, IL-5, IL-13 and Th17 cytokine IL-17 (49, 51, 80). Another study conducted cell co-culture experiments implying that Huaier-induced Th1 differentiation could be mediated by enhanced dendritic cell priming and induction (53). Nonetheless, under the circumstance of immune thrombocytopenia (ITP), Huaier has been found to favor Th2 and Th17 differentiation and inhibit Th1 and Treg differentiation, thereby reversed the imbalanced activation of T lymphocytes in ITP patients (77). Moreover, in rat model of chemically-induced hepatocellular carcinoma, Huaier has been discovered to down-regulate Foxp3+ Treg cells in peripheral blood and facilitated the breakdown of immune tolerance to cancer cells (75). To sum up, Huaier is capable of correcting imbalanced Th1/Th2 and Treg/Th17 differentiation, though the specific modulatory activities are context-dependent and might be different based on the underlying pathophysiological changes.

#### 3.3.2 The effects of Huaier on B lymphocytes

B lymphocytes launch humoral immune responses by producing antigen-specific antibodies to neutralize toxins, block pathogen adherence, activate complements, opsonize organisms for phagocytosis, and enable antibody-dependent cytotoxic attacks against tumor and infected cells by effector killer cells (38). B cell proliferation is an important step in the cascade of humoral immune responses, and Huaier-derived polysaccharides might be capable of stimulating lymphocyte proliferation that are valuable in restoring immunocompromised conditions like cancer (9, 29).

In addition, different isotypes of antibody predominate at different body compartments, exhibit distinct properties and implement differential effects. For example, IgM is the prototype of antibody that responds early to initial attacks and primarily distributes intravascularly; IgG is the main type of antibody in blood and tissues that emerges later after isotype switching; IgA as the secretory type of antibody is involved in mucosa-associated immunity, and IgE is commonly implicated in allergic reaction and parasitic diseases (38, 81). Evidence suggested that Huaier differentially affects antibody production of various isotypes. HQH remarkably increased serum IgA and IgG in pediatric patients with primary nephrotic syndrome (74, 82) and Mycoplasma pneumonia (13). However, the regulatory effect of Huaier on IgM level is undetermined due to contradictory results of existing literature. One meta-analysis found significantly elevated IgM level in Huaier-treated patients with hepatocellular carcinoma (61), which is in agreement with another study investigating the effects of HQH in children with severe Mycoplasma pneumonia (13). Conversely, for pediatric patients with primary nephrotic syndrome, it has been reported by meta-analysis that the regulation of HQH on IgM level was not statistically significant (74) in spite of some positive observation (82). On the other hand, coherent conclusions have been noted that Huaier promoted reduction of serum IgE level in animal models of asthma (49, 80) and parasitic infection (79), indicating amelioration of diseases. Taken together, Huaier stimulates lymphocyte proliferation and refines antibody production, which helps in preventing infection, restraining tumor development and controlling hypersensitive reactions.

### 3.4 Effects of Huaier on myeloid-derived suppressor cells

Myeloid-derived suppressor cells (MDSCs) are one of the major types of immunosuppressive cells in TME that contribute to the formation of immune tolerant milieu in favor of tumor development. By producing immunosuppressive cytokines and expressing inhibitory surface ligands, MDSCs profoundly hamper the activities of effector killer cells and induce anergy and apoptosis of cytotoxic T lymphocytes. In humans and mice, MDSCs consist of polymorphonuclear MDSCs (PMN-MDSCs) and monocytic MDSCs (M-MDSCs). TRMBE, the butanol extract of Huaier, has been found to significantly reduce tumor-infiltrating PMN-MDSCs, with concomitant improvement of cytokine profile and increased CD8+ T cells and NK cells in TME that resulted in restricted tumor growth and prolonged survival of tumor-bearing model mice (17).

### 3.5 Effects of Huaier on signal transduction of chemokines

Duffy antigen receptor for chemokines (DARC) is a member of atypical chemokine binders with absent essential sequence for downstream G-protein coupled signaling (83). As a promiscuous decoy receptor, DARC functionally exerts anti-inflammatory and antiangiogenic activities by decoying miscellaneous CXC and CC chemokines such as CXCL-1, IL-8, CCL-2 and MMP-2. Deficiency in DARC expression is associated with lower allograft survival in organ transplant patients and poor prognosis of various cancers (84). *In vitro* study revealed that Huaier extract enhanced DARC expression in breast cancer cell lines and is related to reduced ligand production like CXCL-1, IL-8, CCL-2 and MMP-2 by cancer cells (84).

## 4 The immunomodulatory effects of Huaier in diseases

### 4.1 Malignancies

In China, Huaier has been employed extensively in clinics for treating a variety of malignancies including hepatocellular cancer, breast cancer, gastrointestinal cancer, etc. Plenty of clinical studies demonstrated its effectiveness in improving survival, preventing recurrence and reducing adverse effects (37, 61, 85). Although the direct inhibitory activities of Huaier on cancer cells has been widely-appreciated (35), emerging evidence suggests an indispensable role of immunomodulation in Huaier-mediated anticancer effects. For example, depletion of NK cells or CD8+ T cells completely abolished the synergistic anticancer effects of the combination treatment of TRMBE and 5-FU (17). Likewise, *in vitro* experiment observed much stronger inhibition on tumor growth by supernatant from Huaier-treated macrophages than by direct Huaier treatment (23). Over decades of research, it has been acknowledged that the surrounding microenvironment of neoplastic cells plays a pivotal role in cancer progression by inducing immune tolerance, facilitating tumor growth and promoting cancer metastasis. Various immune cells are deeply implicated in this process *via* maintaining immunosuppression and provoking angiogenesis. Huaier, with its versatile immune regulatory activities, profoundly modulates the phenotypes of multiple immune cells including tumor-infiltrating macrophages, dendritic cells, NK cells, cytotoxic and helper T cells, which results in the transformation from carcinogenic to tumor inhibitory microenvironment.

Obvious signs of immune enhancement have been reported in numerous studies evaluating clinical values of Huaier in cancer patients. Huaier profoundly boosted peripheral level of NK cells, CD3+ and CD4+ T cells, leading to prominent elevation of the CD4+/CD8+ ratio (37, 61, 72). Marked increasement of serum IL-2 and IgM level has been brought about by the administration of Huaier in patients with hepatocellular cancer (61), indicative of enhanced humoral and cell-mediated immunity. Moreover, Huaier reduced the incidence of myelosuppression in breast cancer patients (72). Synergistic anticancer effects of Huaier have been discovered in combination with chemotherapy, TACE and 125I particle implantation (61), suggesting Huaier as a promising adjuvant therapy for cancer treatment.

For exploring the mechanisms of the immunoregulatory effects of Huaier in cancer treatment, numerous laboratory studies have been conducted and uncovered multifaceted aspects of bioactivities. A study evaluated the effects of Huaier in mouse chemically-induced HCC model, implying the inhibition of Akt/mTOR pathway as a potential factor in mediating the observed therapeutic efficacy of elevated CD4+, CD8+ cells and CD4+/CD8+ ratio together with downregulated Treg, IL-10 and TGF-β (75). Another study found Huaier markedly enhanced the tumoricidal effect of NK cells (65). It has been noted that Huaier increased the expression of the promiscuous decoy receptor DARC on several types of cancer cells, which might improve cancer prognosis by reducing production and abolishing terminal effects of carcinogenic chemokines (84).

Since Huaier effectively modulated T cells and enhanced cell-mediated tumor killing, its activity on dendritic cells has been systemically assessed by several studies. Huaier has been noted to simultaneously elicit increasement of dendritic cells in tumor tissue along with expanded population of CD4+ (53) and CD8+ T cells (42). By activating PI3K/Akt and JNK pathways and suppressing p38 MAPK signaling, Huaier directly promoted the maturation of dendritic cells (53). In addition, Huaier also indirectly enhanced dendritic cell maturation through stimulating the production of DAMPs by tumor cells that subsequently activated dendritic cells (42). Matured dendritic cells markedly enhanced the expression of costimulatory molecules and the production of stimulatory cytokines, thereby dramatically increased their ability in promoting T cell proliferation and inducing Th1 differentiation (42, 53). Thus, dendritic cell is a key mediator whereby Huaier modulates T cells and enhances cytotoxic tumor killing.

Tumor-infiltrating macrophages are usually found detrimental relating to poor prognosis and chemotherapy resistance. The immunosuppressive TME diverts infiltrating macrophages into M2-polarized phenotype that induce immune tolerance, facilitate matrix degradation and promote angiogenesis, favoring tumor growth and metastasis (36, 86, 87). Huaier dose-dependently reduced the infiltration of M2-polarized macrophages in tumor tissue by inhibiting macrophage motility and reducing the release of tumor-derived chemotactic factors (47). By enhancing the signaling of NF-κB/MAPK pathway, Huaier promoted macrophage M1 polarization and stimulated the secretion of antitumor factors like TNF-α, NO and IL-6 (23). Macrophage phagocytic activities were markedly enhanced by Huaier treatment. Furthermore, Huaier potently inhibited macrophage-induced angiogenesis by reducing the production of pro-angiogenic factors such as VEGF, MMP2 and MMP9 (47). Hence, Huaier inhibited tumor growth and metastasis by diverting macrophages into antitumor phenotype and suppressing macrophage pro-angiogenic activities.

### 4.2 Kidney diseases

HQH, the compound preparation of Huaier, has been found effective in the treatment of a series of kidney diseases including IgA nephropathy (IgAN), primary nephrotic syndrome (PNS) and chemotherapy-induced renal damage.

IgAN is a common primary glomerulopathy characterized by abnormal mesangial IgA deposits and mesangial hypercellularity (88). Clinically, IgAN manifests as recurrent hematuria and proteinuria with probable episode of preceding infection, which ultimately leads to ESRD in around 20%~40% of patients within 20 years (89). HQH has been demonstrated to promote the remission of proteinuria and hematuria in patients with mild IgAN (12). The mitigation of IgAN by Huaier treatment is related to inhibition on mesangial proliferation (90) as well as its immunomodulatory activities (91). It has been postulated that altered Th1/Th2 cell balance in favor of Th2 lymphocytes predisposes the onset and exacerbation of IgAN (25). The Th1-inducing activity of Huaier is probably involved in its therapeutic efficacy for IgAN treatment.

Primary nephrotic syndrome is a common cause of chronic kidney disease featuring massive proteinuria, hypoalbuminemia, hyperlipidemia and edema. During the progress of PNS, podocyte damage leads to compromised integrity of the glomerular filtration barrier and massive leakage of proteins into urine (92, 93). Excessive protein deposits in renal tubules injure tubular epithelial cells, which release pro-inflammatory cytokines that prompt the infiltration and activation of monocytes and macrophages resulting in further exacerbation (48). For pediatric patients, minimal change disease (MCD) is the primary cause of PNS. The absence of immune complex and complement deposits in glomeruli and the high responsiveness to steroid and cyclosporin treatment implies the involvement of cell-mediated immunity in the pathogenesis of MCD, which is supported by clinical observations of reduced CD4+ T cells, increased CD8+ T and NK cells, high expression of Th1-associated cytokines (94), impaired Treg quantity and activity (95, 96). Animal experiment consolidated the role of CD4+ T cell depletion in disease progression that leads to increased CD8+ T cell and macrophage infiltration, aggravated histopathological changes and worsening creatinine clearance (97). Although steroid therapy is effective for the majority of patients, approximately 60% experience frequent disease relapses that lead to high risk of treatment side effects and morbidity from complications. Infection is one of the common complications of PNS due to urinary loss of immunoglobulins and complements, impaired lymphocytic function and long-term use of immunosuppressive agents (92). Moreover, steroid-resistant nephrotic syndrome (SRNS) remains a challenge in clinical practice that predicts unfavorable prognosis with 36~50% of patients eventually developing ESRD within a decade (98).

HQH was discovered to regulate multifaceted aspects of PNS. Firstly, the ability of HQH to regulate T cell population benefits the treatment of PNS. It has been discovered that HQH upregulated CD3+ and CD4+ cells and decreased CD8+ cells in PNS patients, thereby corrected the distorted CD4+/CD8+ ratio (74). Further studies are needed to testify whether the modulatory effects of HQH on Th cell differentiation helps in improving aberrant Th1/Th2 and Treg/Th17 balances in PNS. Moreover, HQH showed efficacy in ameliorating the inflammatory milieu of PNS. Pro-inflammatory NF-κB signaling pathway was inhibited in animal ADR-induced nephropathy model by HQH treatment that attenuated p65 and IκBα phosphorylation and nuclear translocation (99). Clinical and experimental studies unveiled that HQH significantly downregulated the expressions of pro-inflammatory cytokines TNF-α, IFN-γ, IL-1β and IL-18 while upregulated both the serum level and immunosuppressive activity of IL-10 in the context of nephropathy (48, 74, 82, 91). In rat ADR-induced nephropathy, HQH ameliorated podocyte injury and reduced infiltration of mononuclear macrophages in renal interstitium (48). Moreover, HQH significantly raised serum IgA, IgG (74) and IgM (82) levels in patients with PNS, which probably contributes to the reduction of infection complications (74). Furthermore, as IL-1β and TNF-α promote glucocorticoid resistance by activating AP-1 that blocks the binding of glucocorticoid-receptor complex with its cognate response element, the IL-1β and TNF-α-lowering effect of HQH suggests its potential in improving glucocorticoid responsiveness of PNS patients (48, 74, 78). The above biological properties all together result in the observed efficacy of HQH for preventing infection, reducing relapse (74) and relieving symptoms (48) in the treatment of PNS.

Chemotherapeutic agents serve as a fundamental pillar in the battle against a variety of cancers. However, accompanying side effects like nephrotoxicity limit the clinical use of many agents. HQH was found to alleviate cisplatin and cyclophosphamide-induced renal damage by exerting versatile anti-inflammatory effects, such as preventing HMGB1 release and nuclear-cytoplasmic translocation, blocking NF-κB nuclear shift, restraining NLRP3 inflammasome signaling, reducing MAPK phosphorylation, diminishing TLR4 expression and decreasing expression of proinflammatory cytokines including IL-6, IL-1β and TNF-α (43, 100). The anti-inflammatory property and the inherent antitumor activity, as well as the minimal toxicity to normal cells and negligible interference on cytotoxic killing of chemotherapy make HQH an excellent candidate of adjuvant treatment for preventing drug-induced nephrotoxicity in cancer patients.

### 4.3 Asthma

Asthma is a chronic inflammatory disorder of the respiratory system featuring variable airway obstruction and bronchial hyperresponsiveness (101). Typical histological alterations include goblet cell hyperplasia, inflammatory cell infiltration and airway remodeling. Derangements of Th cells are deeply involved in the pathogenesis of asthma, in which Th2-secreted cytokines such as IL-4, IL-5 and IL-13 promote the accumulation of eosinophils and Th17-produced IL-17 mediate neutrophil recruitment. Elevated serum IgE level is a common finding of asthma indicative of disease severity (49). In severe asthma patients the phagocytic activity of airway macrophages is remarkably impaired, leading to defects in bacterial clearance and persistence of airway bacteria (102). The therapeutic activities of HQH have been systemically evaluated using ovalbumin-induced asthma model. HQH profoundly reduced the infiltration of various inflammatory cells in airway mucosa and in BALF, including eosinophils, lymphocytes, neutrophils and macrophages. Unbalanced Th cell subsets were effectively corrected by HQH treatment that upregulated Th1/Th2 and Treg/Th17 ratios. Consequently, the distorted cytokine profile was markedly restored by HQH treatment, with significant increasement of Th1- and Treg-associated cytokines IFN-γ and IL-10 as well as downregulation of Th2- and Th17- produced cytokines IL-4, IL-5, IL-13 and IL-17. The levels of TNF-α and TGF-β were also suppressed by the administration of HQH. Furthermore, HQH attenuated serum IgE level and resuscitated the phagocytic activity of alveolar macrophages (49, 51, 80). The immune modulating effects of HQH was comparable and in synergy with that of inhaled glucocorticoids, which justifies the clinical efficacy of HQH in combination with glucocorticoids for the treatment of asthma (11).

### 4.4 Pediatric Mycoplasma pneumonia

The incidence of Mycoplasma pneumonia in children has been witnessed to rising remarkably worldwide, and severe patients suffer from long-term and relapsing course of disease along with accompanying complications. Impeded immunity is a common presentation of severe suffers affecting both cell-mediated and humoral immunity, resulting in high risk of repeated infection. Accelerating recovery from immunocompromised status is a critical task for the treatment of severe Mycoplasma pneumonia, for which HQH showed a satisfactory clinical efficacy. By upregulating serum IgA, IgG and IgM levels, enlarging the population of CD4+ T cells and balancing the ratio of CD4+/CD8+ cells, HQH significantly lowered the rate of reinfection in severe pediatric patients of Mycoplasma pneumonia (13).

### 4.5 Inflammatory bowel diseases

Inflammatory bowel diseases (IBD) are inflammatory diseases of the gastrointestinal tract characterized by chronic and relapsing inflammation. Crohn’s disease and ulcerative colitis are two major types of IBD. Based on available evidence, a hypothesis suggests abnormal immune response against resident intestinal flora as the underlying etiology (103). However, further studies indicated multifactorial etiology, in which altered epithelial barrier function (104), aberrant immune responses (105), dysbiosis of the gut microbiota (106) and genetic disposition (107) cooperate in causing the final illness. Nonetheless, there are no doubt that both innate and adaptive immune system are deeply involved in the pathophysiology. Unbalanced expression profile of proinflammatory and anti-inflammatory cytokines is a fundamental hallmark of IBD pathophysiology (108). Dysregulated immune cell differentiation is another common manifestation featuring derangements of Th cell subsets such as Th1, Th2 and Th17 cells (103). Long-term and recurrent course of IBD could result in serious complications such as intestinal fibrosis (109) and gastrointestinal cancer (110).

Dextran sulfate sodium (DSS) model is a widely adopted chemical-induced animal model for colitis recapitulating IBD pathogenesis (111). A modified model applied the pro-carcinogenic compound azoxymethane (AOM) on top of DSS-induced colitis to simulate colitis-associated colon cancer (CAC) (110). In a recent study, researchers utilized this model to evaluate the therapeutic effects of Huaier aqueous extract on colitis and CAC (112). The results showed that Huaier significantly attenuated gut inflammation as reflected by marked reduction of a panel of cytokines including TNF-α, IL-6, IFN-γ and IL-1β in the colon tissue. Not only was the colon structure preserved better but the tumor load was also greatly reduced in the treatment group, suggesting that Huaier application in IBD might reduce the risk of cancer development partially *via* ameliorating inflammatory milieu of the colon. Detailed research implied reduced phosphorylation of STAT3 by Huaier treatment as the underlying mechanism. Another study demonstrated similar colitis-ameliorating effects of Huaier and attributed its anti-inflammatory activity to impaired inflammasome activation caused by enhancement in NLRP3 degradation *via* the autophagy-lysosome pathway (113).

### 4.6 Juvenile idiopathic arthritis

Juvenile idiopathic arthritis (JIA) is a pediatric rheumatic disease resulted from chronic inflammatory autoimmunity. Systemic involvement is possible which makes JIA a prominent cause of disability and blindness in children. Despite insufficient knowledge on the underlying etiology, it is widely accepted that inflammasomes are deeply involved in the pathogenesis of JIA that trigger the subsequent process of pyroptosis and release of proinflammatory cytokines IL-1β and IL-18. Serum level of IL-18 is indicative of disease activity and associated with the development of macrophage activation syndrome. TNF-α is another key factor for disease progression that stimulates synovial inflammatory response and induces joint destruction. The effects of HQH on JIA has been assessed in mouse collagen-induced arthritis model, which showed effectiveness in relieving general conditions, preventing joint destruction, suppressing synovial expression of GSDMD and caspase-1 as well as reducing serum levels of IL-18 and TNF-α. Hence, HQH might be promising as an adjuvant therapy for the treatment of JIA (114).

### 4.7 Immune thrombocytopenia

Immune thrombocytopenia (ITP) is an autoimmune disorder wherein accelerated platelet destruction and inadequate platelet production result in manifestations like bleeding, low platelet count and fatigue (115). Despite a high response rate to first-line drugs, substantial proportion of ITP patients experience relapses requiring costly or invasive second-line treatment. It is well appreciated that cell-mediated immune reaction plays a crucial role in the pathogenesis of ITP. Overactivated Th1-polarized CD4+ cells produce high levels of pathogenic cytokines such as IL-2, IFN-γ and TNF-α. A preliminary study assessed the effects of Huaier on various biological properties of ITP patients-derived T lymphocytes, showing that Huaier suppressed T cell proliferation, reversed the overactivation of T cells, restored the balance of Th1/Th2 cells and reduced the production of pathogenic cytokines. This study offered a possibility that Huaier might serve as an economical and effective treatment for refractory ITP (77).

### 4.8 Hydatid disease

Hydatid disease (cystic echinococcosis) is a zoonotic disease caused by the parasite Echinococcus granulosus. Larval infection develops into protoscoleces-containing hydatid cysts that occupy internal organs such as livers and lungs (116). Although careful removal of hydatid cysts being a curative therapy, improper surgical management risks the danger of protoscoleces leakage that could lead to catastrophic anaphylaxis (117) or postoperative recurrence. Reinforcing host immunity during perioperative period is a rational way to inhibit protoscoleces growth and prevent recurrence. As an effective immune-enhancing agent, Huaier has been evaluated by scholars for the treatment of hydatid disease in mouse model. Huaier significantly restored CD4+/CD8+ balance, lowered CD8+ T cells and reduced serum IgE level, indicating the effectiveness of Huaier in strengthening cell-mediated immunity and inhibiting hypersensitivity. The combined treatment with Huaier and albendazole produced excellent effect in preventing recurrence, suggesting the clinical value of Huaier in the management of echinococcosis (79).

## 5 Toxicity

Despite being efficacious adjuvant therapies with potent tumoricidal activities, Huaier and its derivations exhibit remarkable low toxicity, as demonstrated by *in vitro*, *in vivo* and clinical research. For example, *in vitro* experiments have shown that the Huaier-derived polysaccharide W-NTRP inhibits cholangiocarcinoma cells with IC50 value of approximately 40~80 μg/mL, while exhibiting no inhibitory effects against normal mouse fibroblast cell L-929 (9). Similar selective cytotoxicity has been observed in breast cancer cells, gastric cancer cells and hepatocellular carcinoma cells, sparing normal breast epithelial cells, gastric epithelial cells and hepatocytes (21, 24, 118). Moreover, the Huaier-derived compound HP-1 not only produced no cell damage to both mouse and human renal tubule epithelial cells, but even provided protective effects against cisplatin-induced injury. These *in vitro* observations were consistent with *in vivo* findings that consecutive intraperitoneal administration of HP-1 at the dose of 60mg/kg was not toxic but instead prevented weight loss and kidney damage induced by cisplatin (32).

Animal experiments provide strong evidence to the safety of Huaier. Various aspects of Huaier-treated animals that could indicate possible toxicity, such as body weight (22, 31, 34, 90, 119–122), behavior (21, 30, 123), appetite and food intake (112, 124), histology of major organs (heart, liver, kidney, lungs, spleen, pancreas and brain) (17, 20, 32, 33, 125), blood assay (20, 23), liver and kidney function (29), and animal survival (112), have been thoroughly examined in numerous studies. However, almost all of these studies reported negligible signs of toxicity in animals, indicating the safety of Huaier in preclinical studies.

High quality clinical trials offer direct evidence for evaluating the toxicity of Huaier in humans. A multicenter randomized controlled trial assigned 696 postoperative hepatocellular carcinoma patients into the Huaier group who received 20g Huaier granule three times a day for 96 weeks (15). Compared with the control group, the most significant drug-related adverse event was diarrhea which reached marginal significance (4.4% in the Huaier group vs. 1.9% in the control group, P=0.0505). Other adverse events were comparable between the two groups. All the reported adverse events were mild and tolerable, suggesting that Huaier is safe for clinical use with low toxicity.

## 6 Discussion

The complexity of the human immune system results in immune-related disorders with sophisticated underlying pathophysiology, sometimes leading to seemingly contradictory manifestations. For example, immunodeficiency and autoimmune diseases were once considered independent sets of diseases representing opposite poles of the immune spectrum; however, with our advances in understanding of the immune system, it has now been accepted that these disorders are actually interconnected and share some common mechanisms (126). The intricate nature of the immune system necessitates the development of novel immunoregulatory therapies that account for the delicate equilibrium of the entire system. Nonetheless, many of the currently available immunoregulatory therapies lack the ability to precisely regulate the entire immune system but instead target specific immune components with narrow therapeutic windows. Consequently, improper delivery of immunotherapies can result in imbalance of the complicated immune system and ensuing adverse events, sometimes may be life-threatening. For example, the improper administration of steroids or immunosuppressants in autoimmune diseases or organ recipients could result in severe infection or malignancies (127–129). Similarly, attempts to boost anti-cancer immune reaction using immune checkpoint inhibitors may lead to immune-related adverse events (irAEs) resembling autoimmune diseases (130). Due to individual variations among patients and the inability to completely grasp their physical status, precise dosing of immunotherapies remains a challenge for clinician. Moreover, many immunotherapies exhibit other inherent side effects unrelated to their immunoregulatory actions, such as the tendency to cause osteoporosis by corticosteroids or hypertension by cyclosporine (131, 132). These shortcomings highlight the risks associated with delivering immunotherapies in clinical practice, despite careful treatment by experienced professionals. Therefore, discovering drugs with minimal side effects, high therapeutic windows and broad immunoregulatory effects is of great value in achieving balance of the immune system.

The potential of Huaier as an effective source for developing innovative medication that meets the aforementioned requirements is noteworthy. Specifically, Huaier exhibits minimal toxicity and side effects, making it a safe option for clinical application. Besides, its broad therapeutic window reduces the risk of overdosing-induced immunodeficiency or autoimmunity. Furthermore, due to its wide-ranging regulatory activities, Huaier functions as a modulator of the entire immune system rather than just a specific target, thereby minimizing the possibility of disrupting the immune network and causing imbalance within the system. Of particular importance is the property exhibited by Huaier, wherein it executes context-dependent regulation, such as inhibiting Th1-differentiation in ITP but promoting Th1-differentiation in asthma. This unique characteristic enables Huaier to restore the immune system to a balanced status, which not only reduces the potential for side effects but also expands the scope of its application.

**Figure 1:**
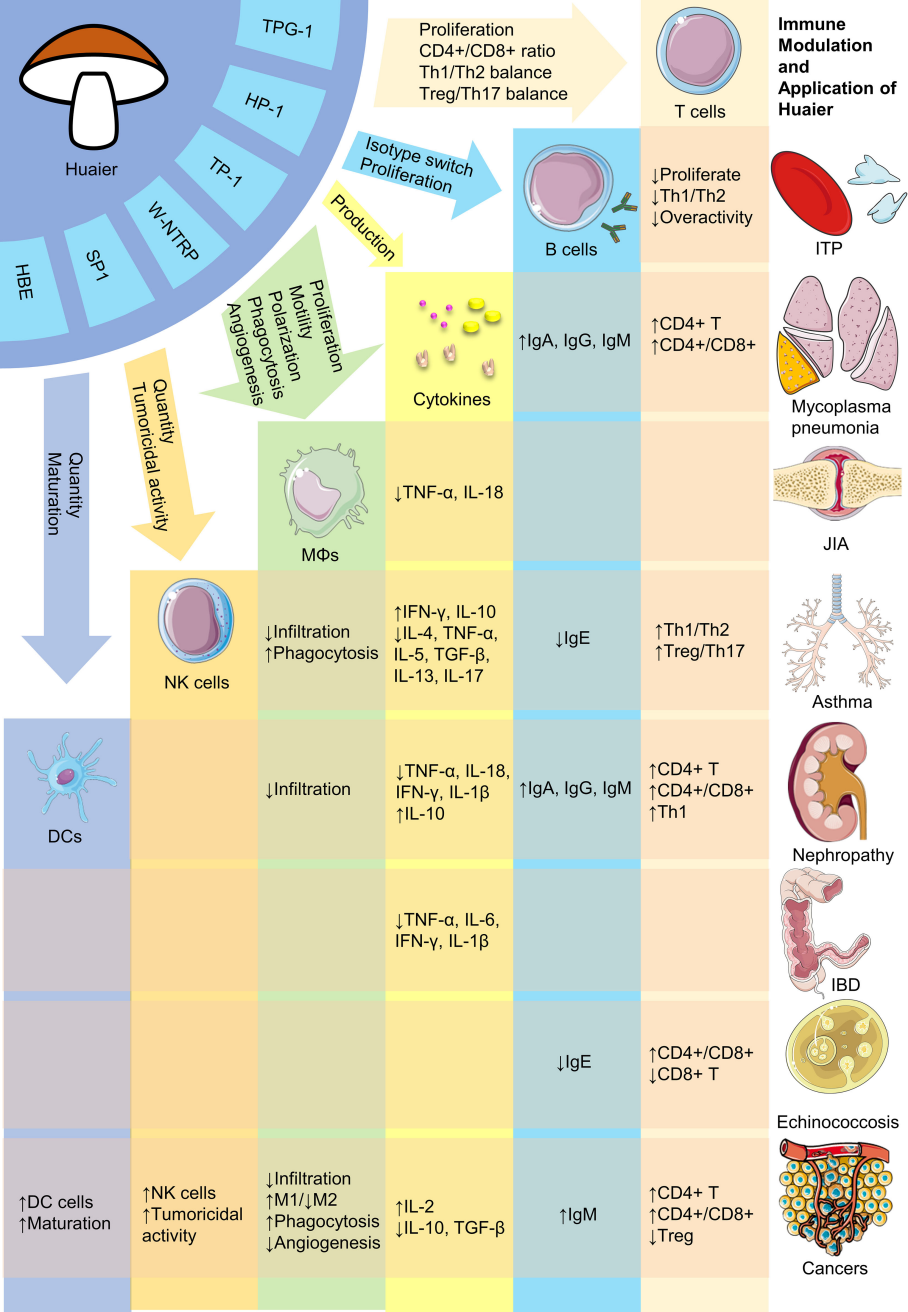
Brief representation of bioactive constituents and immune regulatory activities of Huaier. HBE, Huaier n-butanol extract; DCs, dendritic cells; NK, natural killer; MΦs, macrophages; ITP, immune thrombocytopenia; JIA, juvenile idiopathic arthritis; IBD, inflammatory bowel disease. The figure is partly generated using Servier Medical Art provided by Servier, licensed under a Creative Commons Attribution 3.0 Unported License.

**Figure 2:**
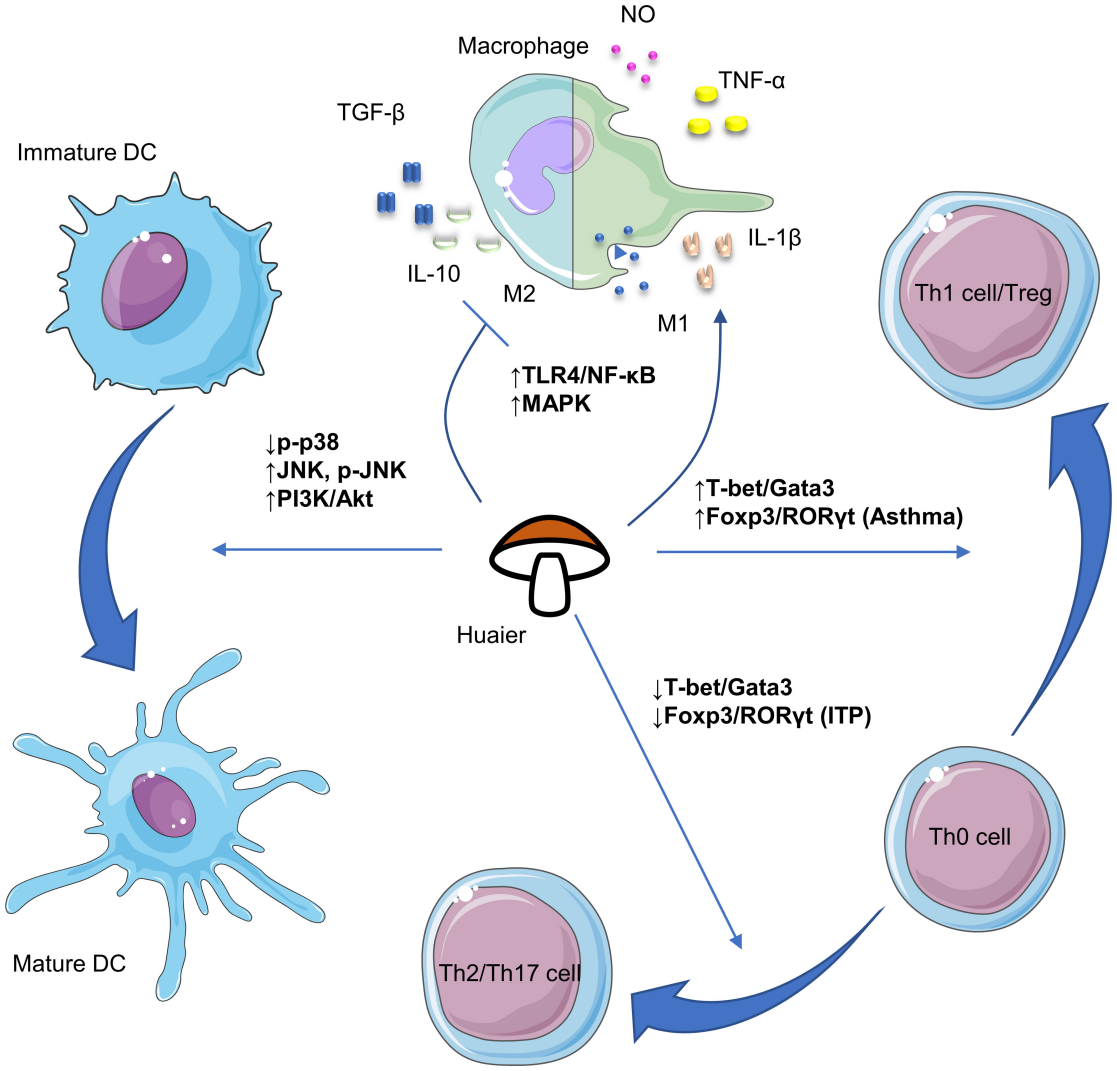
Schematic plots of the signaling pathways that are implicated in the regulation of macrophages, dendritic cells and T cells by Huaier. DCs, dendritic cells; NK, natural killer; ITP, immune thrombocytopenia. The figure is partly generated using Servier Medical Art provided by Servier, licensed under a Creative Commons Attribution 3.0 Unported License.

Despite its numerous advantages, research of Huaier is in a nascent stage and requires substantial future work. Firstly, although efforts have been made to isolate bioactive compounds in Huaier, the primary constituent responsible for its pharmacological effectiveness remains undetermined. Given its complex composition, it is possible that multiple bioactive ingredients with distinct immunoregulatory properties exist, necessitating future research to isolate and clarify their biomedical activities. Secondly, the absence of consensus on the major active components of Huaier impedes determination of their adsorption, distribution, metabolism and excretion, rendering pharmacokinetic research impractical. Thus, it reinforces the importance for identifying the major bioactive compounds. Thirdly, the broad regulatory activities of Huaier make it cumbersome to investigate its possible pharmacological effects. Current available evidence regarding the versatile biological activities of Huaier on various immune cells including macrophages, dendritic cells, NK cells and T lymphocytes have been summarized in **Figure 1**. Also, there are plenty of molecular pathways that are under regulation of Huaier, and part of those that are mentioned in this review have been summarized in **Figure 2**. Although we have dedicated considerable efforts to this area, our comprehension of the immunoregulatory effects of Huaier is still superficial and incomplete. Furthermore, the context-dependent regulation indicates the need to exercise caution when interpreting and extrapolating its effects. Consequently, more extensive efforts are necessary to enhance our understanding of the regulatory activities of Huaier.

In summary, the traditional Chinese medicine Huaier has been recently discovered to exhibit versatile immunomodulatory activities, leading to its increasing clinical use for the treatment of a variety of immune-related disorders such as malignancies, nephropathy, asthma and pneumonia. Given its low toxicity and limited side effects, Huaier and its derivatives hold promise as an efficacious therapy for a wide range of diseases, including allergy, immunodeficiency and autoimmunity. Future research is warranted to fully leverage the biomedical potential of Huaier.

## Author contributions

HL systemically conducted literature collection and viewpoint summary, and wrote the draft of the manuscript. ZW checked the eligibility of collected literature and the accuracy of extracted viewpoints, revised the manuscript, and plotted the schematic diagram. All authors contributed to the article and approved the submitted version.
